# Pre-pubertal oocytes harbor altered histone modifications and chromatin configuration

**DOI:** 10.3389/fcell.2022.1060440

**Published:** 2023-01-10

**Authors:** Pe’era Wasserzug Pash, Gilad Karavani, Eli Reich, Lital Zecharyahu, Zehava Kay, Dvora Bauman, Talya Mordechai-Daniel, Tal Imbar, Michael Klutstein

**Affiliations:** ^1^ Institute of Biomedical and Oral research, Faculty of Dental Medicine, The Hebrew University of Jerusalem, Jerusalem, Israel; ^2^ Fertility Preservation Service, Department of Obstetrics and Gynecology, Hadassah Ein Kerem Medical Center and Faculty of Medicine, The Hebrew University of Jerusalem, Jerusalem, Israel

**Keywords:** oocytes, puberty, ovary, chromatin, histone modificaitons, FSH

## Abstract

Pre-pubertal oocytes are still dormant. They are arrested in a GV state and do not undergo meiotic divisions naturally. A multitude of molecular pathways are changed and triggered upon initiation of puberty. It is not yet clear which epigenetic events occur in oocytes upon pubertal transition, and how significant these epigenetic events may be. We evaluated epigenetic marker levels in mouse pre-pubertal and post-pubertal female oocytes. In addition, we evaluated H3K9me2 levels in human oocytes collected from fertility preservation patients, comparing the levels between pre-pubertal patients and post-pubertal patients. The chromatin structure shows a lower number of chromocenters in mouse post-pubertal oocytes in comparison to pre-pubertal oocytes. All heterochromatin marker levels checked (H3K9me2, H3K27me3, H4K20me1) significantly rise across the pubertal transition. Euchromatin markers vary in their behavior. While H3K4me3 levels rise with the pubertal transition, H3K27Ac levels decrease with the pubertal transition. Treatment with SRT1720 [histone deacetylase (HDAC) activator] or overexpression of heterochromatin factors does not lead to increased heterochromatin in pre-pubertal oocytes. However, treatment of pre-pubertal oocytes with follicle-stimulating hormone (FSH) for 24 h - changes their chromatin structure to a post-pubertal configuration, lowers the number of chromocenters and elevates their histone methylation levels, showing that hormones play a key role in chromatin regulation of pubertal transition. Our work shows that pubertal transition leads to reorganization of oocyte chromatin and elevation of histone methylation levels, thus advancing oocyte developmental phenotype. These results provide the basis for finding conditions for *in-vitro* maturation of pre-pubertal oocytes, mainly needed to artificially mature oocytes of young cancer survivors for fertility preservation purposes.

## Introduction

To achieve oocyte maturation, the activation of a whole array of epigenetic pathways and chromatin factors is required. Oocyte growth, development, and meiotic function are characterized by a unique epigenetic environment, designed to advance oocyte maturation, and prepare the ground for the transformation into an embryo. Histone composition and chemical modifications are known to contribute to oocytes function. For example, oocyte-specific histone variants such as H3.3 and H1foo are required for proper oocytes maturation, as well as paternal chromatin organization after fertilization (Wen et al., 2014) (Torres-Padilla et al., 2006) (Funaya et al., 2022). Another prominent example is that histone modification patterns also play a vital role in oocyte development. Histone methylations are involved in parental imprinting pattern establishment and accompany the transition from the immature non-surrounded nucleolus chromatin configuration to the mature surrounded nucleolus form (Kageyama et al., 2007) (Bonnet-Garnier et al., 2012) (Stewart et al., 2015). Moreover, histone post-translational modifications (PTM) modulate transcriptional activity (Jenuwein and Allis, 2001) (Li et al., 2007) in the oocyte, and play an important role in kinetochore establishment and chromosome segregation as part of their meiotic function (Gonzalez-Barrios et al., 2012) (Bergmann et al., 2011). Changes in the pattern of histone PTMs and DNA methylation are characterized as part of oogenesis and oocyte maturation (Wasserzug-Pash and Klutstein, 2019), as well as a response to stress, stimulation, and aging (Seong et al., 2011). Impaired histone modification is associated with loss of functionality and meiotic anomalies in oocytes, including impaired gene expression and DNA damage (Wasserzug-Pash et al., 2022) (Wang et al., 2006) (Bao et al., 2000). Therefore-the epigenetic modifications on histones can affect oocyte maturation and control its efficiency.

The importance of histone PTMs is currently described mainly throughout oocytes developmental progression, their follicular growth and meiotic resumption. However, most oocytes will never reach this stage, and almost all oocytes exist long before their growth is initiated. Oocytes are generated during embryonic development, and then remain dormant until they are triggered into growth by hormonal signals. Within the growing oocytes population, almost all the oocytes will be eliminated before meiotic resumption, and only a small number will be ovulated (McGee and Hsueh, 2000).

Thus, our current understanding of the role of histone PTMs in arrested oocytes - before they resume meiosis-is lacking. We also lack information on the stability of histone PTMs during different stages of female life in dormant oocytes. Our previous work has revealed that aged oocytes (9 months old mouse females) show a loss of heterochromatic histone modifications (H3K9me2 and H3K27me3) and as a result-suffer activation of retrotransposons and DNA damage (Wasserzug-Pash et al., 2022). As fertility preservation procedures have grown more and more common in recent years, it has become crucial to understand the cellular activity of oocytes during the pre-pubertal stage of life. We thus sought to implement the same tools as used in our previous work to explore histone PTMs in pre-pubertal oocytes.

Improvements in cancer diagnostics and therapies, resulting in increased survival rates of children. Currently, 80% of diagnosed children achieve 5-year survival (Ward et al., 2014). Thus, childhood cancer survivors’ development and life quality are gaining importance, including a growing interest in the reproduction capability of these patients. Fertility preservation procedures in sexually mature women undergoing gonadotoxic treatment are well-established and relatively effective. However, in pre-pubertal patients, fertility preservation procedures are usually limited to a few clinical solutions. The first solution is ovarian tissue cryopreservation (OTC) and re-introduction of the cryopreserved tissue upon remission. Another viable option is *ex-vivo* oocyte *in-vitro* maturation (IVM) from the tissue or OTC medium (Anderson and Wallace, 2011) (Wang et al., 2016) (Algarroba et al., 2018). Moreover, it is noteworthy that the option of OTC and re-introduction of tissue is limited due to the risk of re-introduction of transformed cells. Therefore, IVM of immature oocytes is the only viable option for fertility preservation in some cases such as leukemia.

However, IVM of pre-pubertal oocytes is problematic. All current evidence implies that pre-pubertal oocytes have impaired reproductive function. In our previous work, we showed that in *ex-vivo* IVM of oocytes from fertility preservation patients, pre-pubertal oocytes perform poorly, and have low maturation efficiency (Karavani et al., 2021) (Karavani et al., 2019). These oocytes were also shown to have increased aneuploidy rates (Gruhn et al., 2019). However, more information is needed regarding molecular and biological mechanisms involved in pre-pubertal oocyte dysfunctionality.

To provide valuable insight into the function of the pre-pubertal oocytes, we examined their chromatin organization and histone modifications, and how those change during puberty. We also explored possible mechanisms that drive such changes, including hormonal stimulation.

Hormones are known effectors of epigenetic states in many kinds of cells (Zhang and Ho, 2011) (Heemers et al., 2007) (Reizel et al., 2015) (Zannas, 2021). Female sex hormones and specifically gonadotropins also have epigenetic effects. Progesterone and estrogen regulate DNA methyltransferases expression and activity (Muller et al., 2003) (Yamagata et al., 2009) (Cui et al., 2009), as well as histone deacetylase function (Saji et al., 2005). FSH is a known mediator of epigenetic changes during spermatogenesis (Zannas, 2021) (Ankolkar and Balasinor, 2016) (Lujan et al., 2019) and was shown to induce DNA methylation changes in oocytes (Huo et al., 2020). Therefore, different features of the oocyte epigenome are expected to change during puberty because of hormonal changes.

In all eukaryotic cells, chromatin is composed of two main configurations: heterochromatin, the tightly compacted, transcriptionally silent sequence regions, and euchromatin, the dispersed, transcriptionally active sequences. Histone post-translational modifications (PTMs) and epigenetic features are central aspects of chromatin identity and function (Li et al., 2007). In general, histone acetylation forms an open and dispersed nucleosome composition that is associated with euchromatin. Histone methylations are more diverse in their roles and are highly context-dependent. To detect heterochromatin in the present study, H3K9 methylation and H3K27 methylation are used, which are known to be associated with silent transcription and nucleosome compaction. In contrast, H3K4 methylation, is associated with actively transcribing promoters and euchromatin and thus is used in the present study to detect euchromatic regions (Peng et al., 2021) (Zhang et al., 2021) (Hsu et al., 2021).

A recent report characterized the quality and maturation ability of mouse pre-pubertal oocytes at different ages after birth (Kusuhara et al., 2021), producing detailed information about the exact timing of multiple aspects of puberty in CD-1 mouse oocytes. The report shows that, as in human pre-pubertal oocytes, mouse pre-pubertal oocytes fail to efficiently mature *in-vitro*, and have reduced quality in multiple aspects compared to post-pubertal oocytes. This model provides a strong basis for continued investigation of pre-pubertal oocytes in a mouse model, and enables testing findings on clinically available human material.

We provide a closer look at the status of histone modifications of human and mouse pre-pubertal oocytes. With puberty, a drastic chromatin re-organization event occurs in both species. We show that this event is partially mediated by the follicle-stimulating hormone (FSH) on the oocyte. Exposure to FSH and activation of this chromatin remodeling process results in a more advanced histone modification phenotype, demonstrating the importance of nuclear changes in oocytes during the pubertal transition.

## Results

### Pre-pubertal mouse oocytes mature poorly *in-vitro*

To validate our mouse oocyte *in-vitro* maturation (IVM) system in C57BL/6 mice, IVM was performed on oocytes retrieved from ovaries of 3 weeks old (pre-pubertal), and 7–13 weeks old (post-pubertal) females. Successful maturation was defined by polar body extrusion with visible and defined segregation of chromatin into two separate entities. (Observed by staining with Hoechst). Our results confirmed previous observations (Karavani et al., 2021) (Karavani et al., 2019) (Kusuhara et al., 2021) reporting that post-pubertal oocytes present higher rates of successful maturation than pre-pubertal oocytes (Supplementary Figure S1), and lower rates of cells that underwent meiosis but presented abnormal segregation (Supplementary Figure S1).

While pre-pubertal oocytes grow only through the continuous, hormone-independent follicular recruitment process, post-pubertal oocytes have an additional phase of FSH-triggered follicular growth (McGee and Hsueh, 2000). Based on this knowledge, it is expected that the size range of the pre-pubertal oocyte retrieved from the ovary will be smaller than post-pubertal oocytes, as observed. The post-pubertal oocyte pool also includes a population of large mature oocytes not found in pre-pubertal ovaries. These represent the maturing oocytes found within the post-pubertal ovary. Notably, oocytes from pre-pubertal mice were of smaller size. This observation was consistent in the GV stage, and after IVM (Supplementary Figures S2A–C).

### Nucleus architecture is re-organized in mouse oocytes across the pubertal transition

**FIGURE 1:**
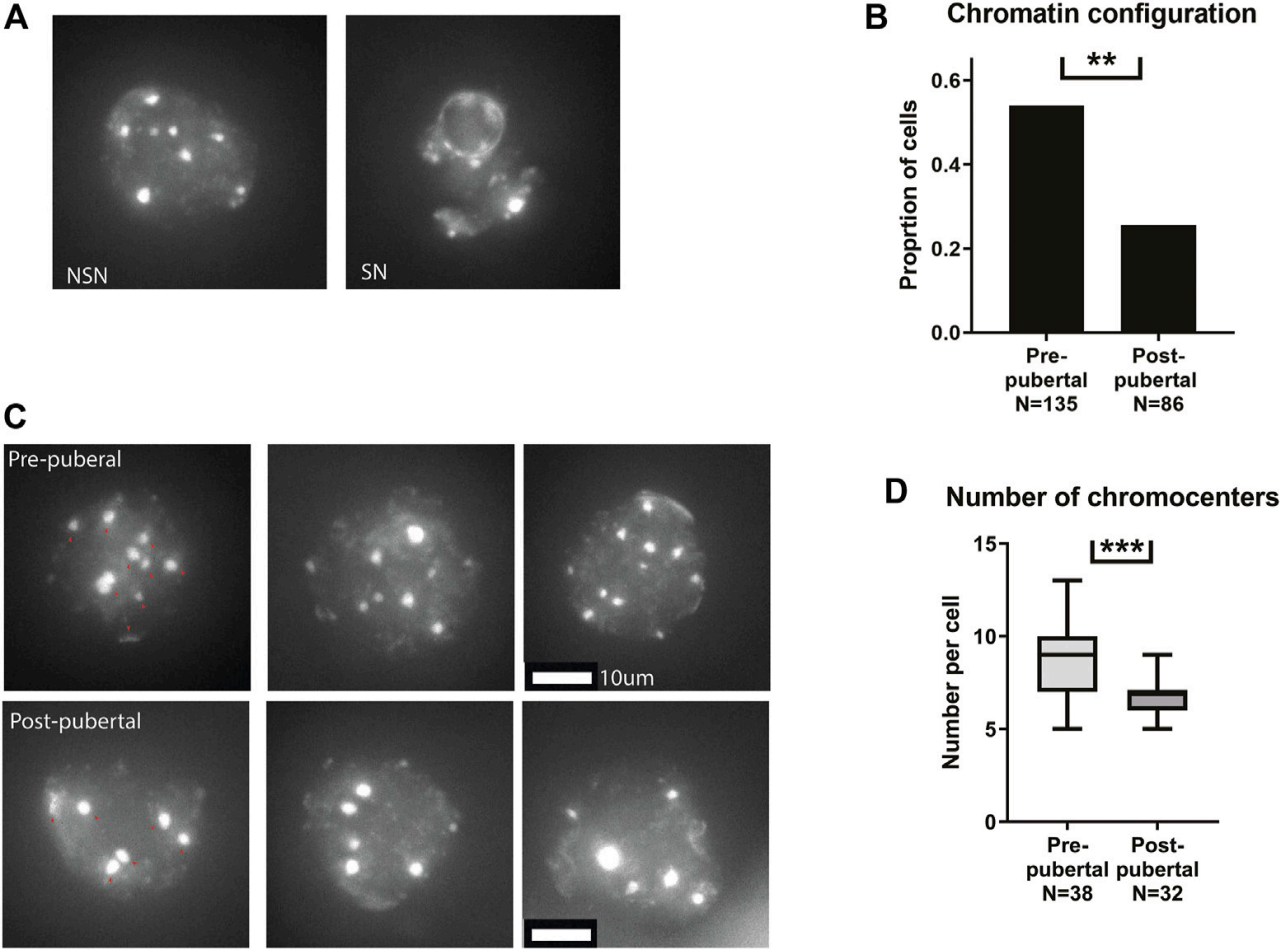
Pre-pubertal mouse oocytes present poor meiotic function and aberrant chromatin organization: **(A)** Meiotically arrested oocytes were collected from the same populations, and their chromatin condensation rate was measured. Examples of SN and NSN configurations are presented. **(B)** Before puberty, oocytes presented a more common appearance of the immature NSN configuration (Z test *p* = .00604, N_pre-pubertal_ = 8, N_post-pubertal_ = 5). **(C)** Examples of pre-pubertal and post-pubertal NSN oocytes, with red arrows marking Chromocenters. **(D)** Within NSN oocytes, a less condensed nuclear organization was present before puberty, with an increase in the number of chromocenters. (Welch’s *T*-test *p* = .0006 Npre-pubertal = 8, Npost-pubertal = 5).

After validating the phenotype in our mouse model, we turned to investigate nuclear organization of pre-pubertal oocytes. Nuclear organization in oocytes is well characterized, and the post-pubertal adult nucleus configuration is associated with proper cellular maturation and meiotic progression. The transition from the non-surrounded nucleolus (NSN) to the surrounded nucleolus (SN) nuclear configurations is an important landmark in the oocyte biology. NSN oocytes are considered less competent in terms of meiotic function and cellular growth (Tan et al., 2009), and achievement of SN configuration is a marker of cellular functionality. Thus we examined nuclear configuration in pre-pubertal C57BL/6 mouse oocytes in GV stage immediately after oocyte extraction from the ovaries, i.e., meiotically arrested oocytes from antral or bigger follicles. Oocytes from pre-pubertal females presented significantly higher rates of NSN-configured oocytes (Figures 1A, B). We then focused on oocytes with NSN nuclear organization. Specifically, we focused on chromocenter clustering in NSN cells comparing pre- and post-pubertal oocytes. Chromocenters are dense loci of constitutive heterochromatin regions, and their arrangement drastically changes during mouse oocyte growth (Bonnet-Garnier et al., 2012). The clustering of chromocenters takes place as part of oocyte growth and NSN to SN transition. Thus, chromocenter loci were counted in NSN cells from pre-pubertal and post-pubertal females. Oocytes from pre-pubertal mice presented a higher number of chromocenters in the nucleus showing a less advanced configuration (Figures 1C, D). Taken together, these results demonstrate pre-pubertal specific unique nuclear organization. Specifically, pre-pubertal oocytes nuclei are organized in patterns that are considered less ready to enter meiosis.

### Histone modification patterns are reformed across the pubertal transition

**FIGURE 2:**
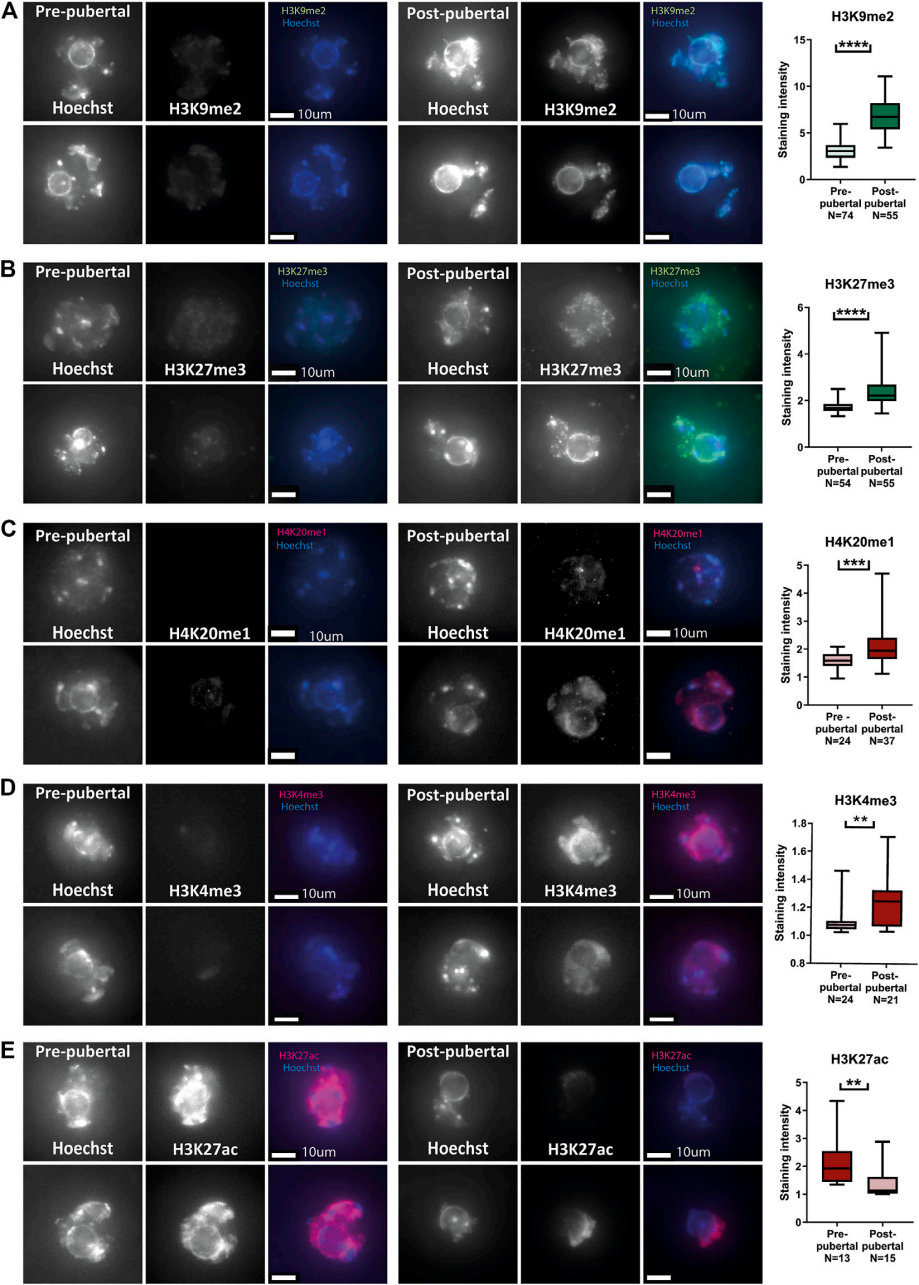
Histone post-translational modifications pattern changes during pubertal transition in mouse oocytes: *In situ* staining was performed on pre-pubertal and post-pubertal oocytes, for a panel of histone modifications. **(A–E)** Before puberty, methylation levels were lower in heterochromatic markers H3K9me2 **(A)** (Welch’s -test *p* < .0001, N_pre-pubertal_ = 3, N_post-pubertal_ = 3), H3K27me3 **(B)** (Welch’s T-test *p* < .0001, N_pre-pubertal_ = 4, N_post-pubertal_ = 4), and H4K20me1 **(C)** (Welch’s T-test *p* = .0001 N_pre-pubertal_ = 3, N_post-pubertal_ = 4). Lower methylation levels were observed also in the euchromatic H3K4me3 marker **(D)** (MW test *p* = .0027 N_pre-pubertal_ = 4, N_post-pubertal_ = 4). Acetylation levels, in contrast, were higher before puberty, as measured by staining for H3K27ac **(E)** (MW test *p* = .0064 Npre-pubertal = 3, Npost-pubertal = 5).

In addition to investigating chromatin structure, we investigated histone post-translational modifications (PTM) patterns in pre-pubertal oocytes. Immuno-staining for a panel of modifications was performed, including both euchromatic and heterochromatic modifications. Pre-pubertal oocytes presented lower heterochromatin markers staining than mature oocytes, for three different markers: H3K9me2, H3K27me3 and H4K20me1 (Figures 2A–C). The staining pattern for euchromatin markers was more variable: H3K4me3 staining had lower intensity in pre-pubertal oocytes compared to post-pubertal oocytes (Figure 2D). However, H3K27Ac staining was significantly higher in pre-pubertal oocytes compared to post-pubertal oocytes (Figure 2E). To determine if oocyte size difference between the age groups influenced histone modifications, we analyzed the levels of histone PTMs in oocytes from similar sizes of both age groups (“overlapping cells,” Supplementary Figure S2C). H3K9me2 staining showed higher levels in post-pubertal oocytes than in pre-pubertal oocytes of the same size (Supplementary Figure S2D). This result indicates that the changes in histone modifications occur during pubertal transition independent of oocyte size.

### Changes that occur in H3K9me2 during pubertal transition are also observed in human oocytes

To investigate the conservation of our observations from mouse to human oocytes, we performed similar experiments in human oocytes from OTC fertility preservation procedures. Having limited access to human oocytes, we chose one representative histone mark that showed changes in mouse oocytes. H3K9me2 is the canonical constitutive heterochromatin marker. It is localized mostly to DNA regions that are tightly repressed like genomic repeats and retroviral sequences, and is spread along centromeres, telomeres, and silenced rDNA loci (Saksouk et al., 2015). Therefore, changes to this marker’s levels may represent major epigenetic remodeling events.

**FIGURE 3:**
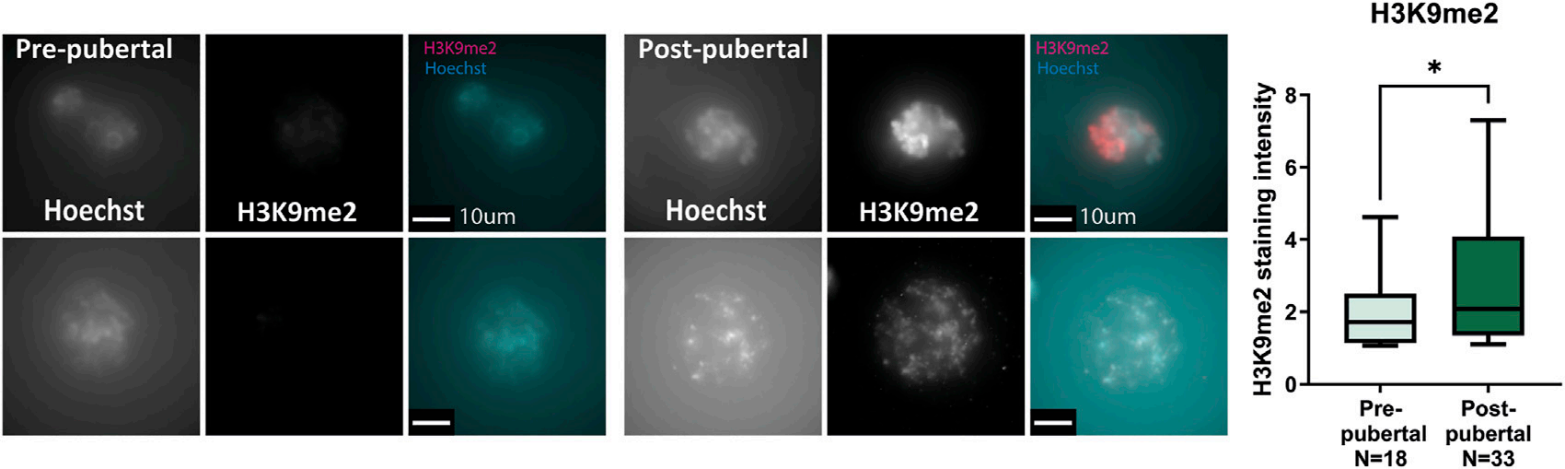
Human oocytes undergo H3K9me2 changes with puberty: Pre-pubertal and mature oocytes were collected from disposed medium after OTC procedure, followed by *in situ* staining for H3K9me2. Consistent with our previous results in mouse oocytes, post-pubertal oocytes presented increased H3K9me2 staining compared to pre-pubertal oocytes. (MW test pv = .048).

Human oocytes from OTC medium were collected, fixed after 24 h in culture, and stained for H3K9me2 (see Methods). As in mouse oocytes, staining intensity of human oocytes from post-pubertal patients was significantly higher than oocytes from pre-pubertal patients. Interestingly, no significant difference was found in the size of oocytes retrieved from pre-pubertal and post-pubertal patients, possibly due to the extraction methods employed during OTC, which may favor oocytes of specific sizes (Figure 3; Supplementary Figure S3; Supplementary Table S1). The notable difference in H3K9me2 levels between pre-pubertal and post-pubertal oocytes is consistent with our findings of chromatin re-organization with puberty in mice and suggests a chromatin re-organization event that is initiated across puberty in mammalian oocytes. To better understand this event and its mechanism, we have investigated the effect of chromatin-modifying drugs on pre-pubertal oocytes chromatin.

### Retrotransposons show increased mRNA transcription but no increase in protein levels in prepubertal oocytes

**FIGURE 4:**
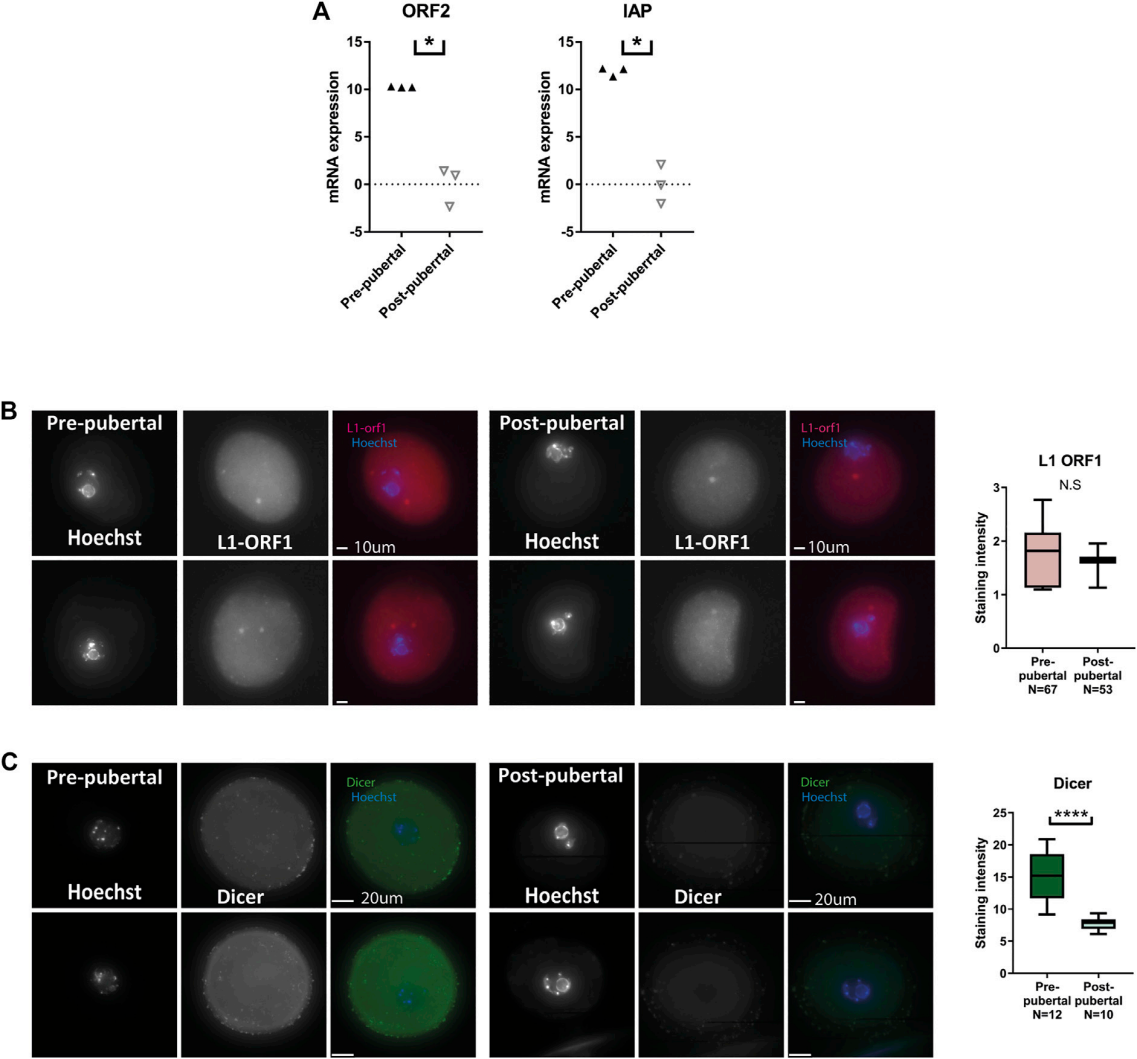
Retrotransposon mRNA expression is increased in pre-pubertal mouse oocytes but protein levels remain in check: **(A)** qRT-PCR for retrotransposon mRNA sequences was performed on cDNA from pre-pubertal and post-pubertal oocytes. An increase in retrotransposon transcription was observed in IAP (MW *p* = .05) and ORF2 (MW *p* = .05, N_pre-pubertal_ = 8, _-pubertal_ = 10) sequences. **(B)** To assess retrotransposon protein levels, *in situ* staining was performed for LINE1 retrotransposon protein L1-ORF1 in pre-pubertal and post-pubertal oocytes. No significant difference was observed in mean staining intensity between the age groups (Welch’s T-test *p* = .603, N_pre-pubertal_ = 3, N_post-pubertal_ = 3). **(C)** A higher level of Dicer was observed in pre-pubertal oocytes by *in-situ* staining (MW *p* < .0001, N_pre-pubertal_ = 4, N_post-pubertal_ = 4), which could explain the difference between mRNA expression and protein expression results.

In our previous work, we showed that heterochromatin loss in oocytes results in an elevation of retrotransposon RNA and protein expression, an increase in DNA damage, and loss of cellular functionality (Wasserzug-Pash et al., 2022). Following our observations of lower levels of heterochromatin histone modifications in pre-pubertal oocytes, we examined retrotransposon expression in those cells. qRT-PCR was performed for retrotransposon sequences. Indeed, higher levels of retrotransposon mRNA were detected (Figure 4A). However, immuno-staining for Line 1 open reading frame 1 protein (L1-ORF1p), a retrotransposon protein, produced no statistically significant difference between oocyte populations, although variability in staining level was much larger in pre-pubertal oocytes (Figure 4B). Increased RNA expression does not always produce a parallel increase in protein levels. When this occurs-it is mainly due to the activity of post-transcriptional regulatory pathways. One of the major players in post-transcriptional regulatory pathways is the protein Dicer, a protein with RNAse activity and a key player in mRNA degradation, RNAi and micro-RNA pathways. Therefore, we stained the two oocyte populations for Dicer. A higher level of Dicer was apparent in pre-pubertal oocytes (Figure 3C), suggesting that post-transcriptional regulatory pathways are highly active in these oocytes. The increased activity of post-transcriptional regulation may explain the gap between the rise in retrotransposon mRNA in pre-pubertal oocytes, and the lack of changes in retrotransposon protein levels. Dicer activity might target retrotransposon transcripts and prevent them from being translated into protein in pre-pubertal oocytes, thus saving these oocytes from resulting DNA damage.

### Heterochromatin histone modifications in pre-pubertal oocytes are stable

To have a more detailed understanding of the role of histone modification patterns on the unique properties of pre-pubertal oocytes, we attempted an intervention approach in the activity of histone-modifying enzymes. We focused on heterochromatin-modifying enzymes, using chemical and genetic manipulations. This strategy has been extensively used in other types of cells (Zhang et al., 2019) (Kong et al., 2022) (Kopytko et al., 2021) (Farahi et al., 2022) and in our lab on oocytes (Wasserzug-Pash et al., 2022).

**FIGURE 5:**
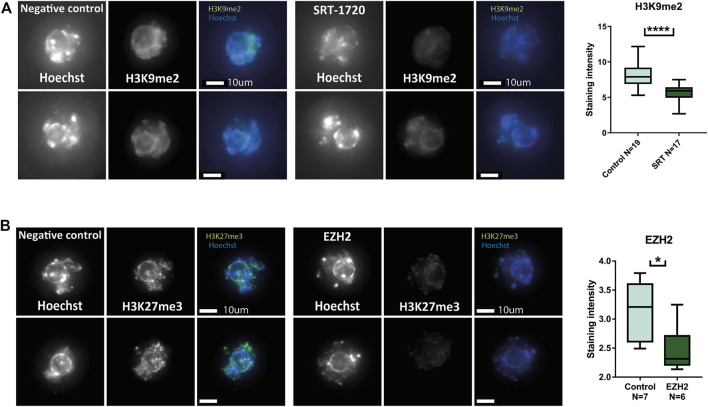
Heterochromatin-enhancing compounds do not increase heterochromatic modifications in pre-pubertal oocytes: Oocytes were collected and treated with heterochromatin-enhancing compounds. Prolonged culture with SRT-1720 treatment successfully produced an increase in methylation levels of aged oocytes in our previous publication. **(A)** A similar assay was performed on pre-pubertal oocytes, followed by *in-situ* staining for H3K9me2. However, in pre-pubertal oocytes SRT-1720 presented decreased methylation levels compared to untreated controls (MW *p* < .0001, N_animal_ = 4). Genetic intervention was performed as well, using plasmid electroporation. EZH2 electroporation resulted in increased H3K27me3 in a previous publication. **(B)** Pre-pubertal mouse oocytes electroporated with EZH2 plasmid were stained for H3K27me3 after prolonged culture. The treated group presented lower levels of staining compared to empty-medium electroporated controls (MW *p* < .0111, N_animal_ = 3).

Due to the increased acetylation of H3K27 in pre-pubertal oocytes, we targeted histone acetyl-transferase activity, by targeting SIRT1. The histone deacetylase SIRT1 is essential for maintaining the epigenetic profile that ensures oocyte viability. In addition to its specific contribution to oocyte histone modification establishment, SIRT1 also targets H3K9 and H3K27 histone tail residues that presented different modification patterns in our experiments (Wasserzug-Pash et al., 2022) (Chen et al., 2019) (Nevoral et al., 2019). Thus, we attempted activating SIRT1 using the chemical activator molecule SRT-1720. In our previous work, we showed that treatment with SRT-1720 increased heterochromatin levels in reproductively aged oocytes (Wasserzug-Pash et al., 2022). Surprisingly, treatment of pre-pubertal oocytes did not increase H3K9me2 levels, but instead-reduced them (Figure 5A). In addition, we attempted a genetic intervention by overexpressing a heterochromatin enzyme in pre-pubertal oocytes. Enhancer Of Zeste 2 - EZH2 is a component of the polycomb repressive complex 2 (PRC2) complex. EZH2 methylates H3K27 histone tail residues, which results in transcriptional repression over the area of modification. In a similar manner to SRT-1720 treatment, our previous work shows that overexpression of this enzyme resulted in increase of H3K27me3 in reproductively aged oocytes (Wasserzug-Pash et al., 2022). Our overexpression method requires active transcription in pre-pubertal GV oocytes. To validate active transcription in these cells, we stained them for 5’ RSS-CTD (repeat YSPTSPS phospho S5 on C-terminal domain) RNA polymerase II, a marker for active transcription. A positive signal was observed in pre-pubertal oocytes, with no significant difference between pre-pubertal and post-pubertal oocytes (Supplementary Figure S4A). To further validate successful electroporation and transcription of mRNA from the plasmid, we overexpressed GFP in pre-pubertal oocytes (Supplementary Figure S4B).

Finally, when overexpression of EZH2 was performed in pre-pubertal oocytes, it resulted in a decrease in H3K27me3 staining instead of an increase as previously achieved in reproductively aged oocytes (Figure 5B).

These results suggest that the pattern of histone modifications in pre-pubertal oocytes is actively preserved, in contrast to post-pubertal oocytes that readily responded to epigenetic intervention (Wasserzug-Pash et al., 2022). This could stem from inaccessible chromatin configuration in pre-pubertal oocytes or from the existence of an active enzymatic mechanism that preserves histone tail demethylation in pre-pubertal oocytes.

### Treatment of mouse pre-pubertal oocytes with FSH initiates changes in histone modifications

We searched for a mechanism that could mediate the natural transition of histone modification in oocytes during puberty. Hormonal activity could be involved in initiating nuclear changes during pubertal transition (see introduction). An important candidate for a hormone that may perform this function is the gonadotropin, follicle-stimulating hormone (FSH). FSH levels are known to rise significantly with the pubertal transition (Latronico et al., 2016) (Neely et al., 1995). Furthermore, FSH is known to be able to induce chromatin changes in spermatocytes and oocytes and other cell types such as adipocytes when administered systemically (Kumar et al., 1991) (Verdi et al., 2021) (Chen et al., 2020) (Soares et al., 2020). Therefore, we sought to investigate a possible role of FSH by mimicking, *in-vitro*, the effect of FSH during the pubertal transition.

**FIGURE 6:**
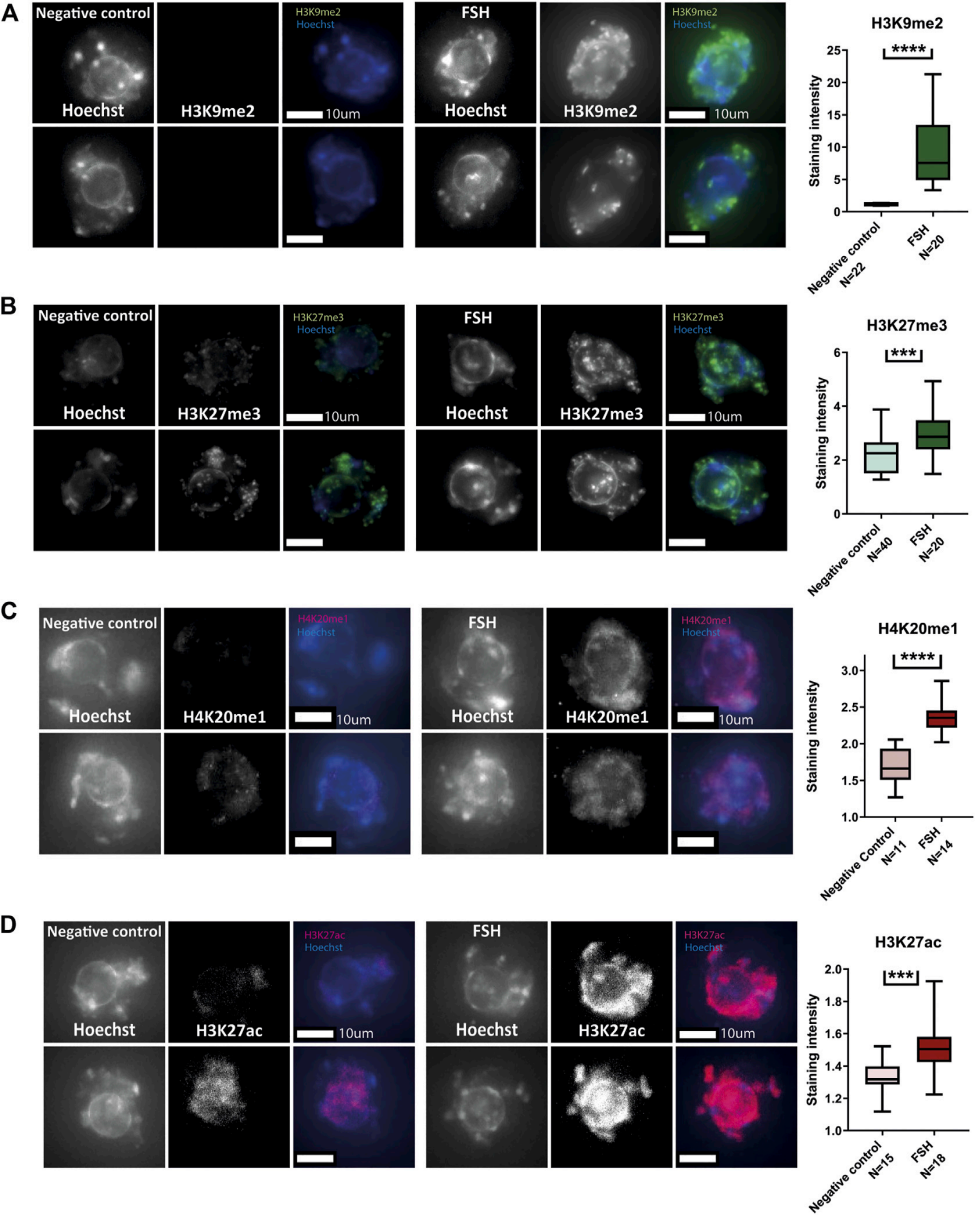
*In-vitro* treatment of pre-pubertal oocytes with FSH initiates histone modification changes: **(A–D)** Meiotically arrested oocytes from pre-pubertal mice underwent prolonged culture in FSH and stained for a panel of histone modification markers. Methylation levels were increased as a result of FSH exposure-as shown for H3K9me2 **(A)** (T-test *p* < .0001, Nanimal = 4), H3K27me3 **(B)** (*t*-test *p* = .0001, Nanimal = 4) and H4K20me1**(C)** (MW *p* < .0001, Nanimal = 3). Acetylation levels were increased as well, as shown by staining for H3K27ac **(D)** (MW *p* < .0001, Nanimal = 3).

Pre-pubertal mouse oocytes were collected and exposed in culture for 24 h to FSH-supplemented medium, followed by fixation and immune staining for a panel of histone PTMs. FSH-treated oocytes presented histone methylation patterns that were similar to post-pubertal oocytes. H3K9me2 H3K27me3 and H4K20me1(Figures 6A–C), were increased after treatment. However, histone acetylation did not follow the same trend as histone methylation and showed a further increase in signal after FSH treatment, in contrast to a marked decrease during pubertal transition (H3K27ac, Figure 6D). This result shows that FSH has a significant role to initiate nuclear changes during pubertal transition. However, FSH is not sufficient to reach the full array of post-pubertal histone PTM patterns, which may require additional factors and signaling.

### FSH treatment of mouse pre-pubertal oocytes initiates changes in chromatin organization and alters meiotic and cellular functions

**FIGURE 7:**
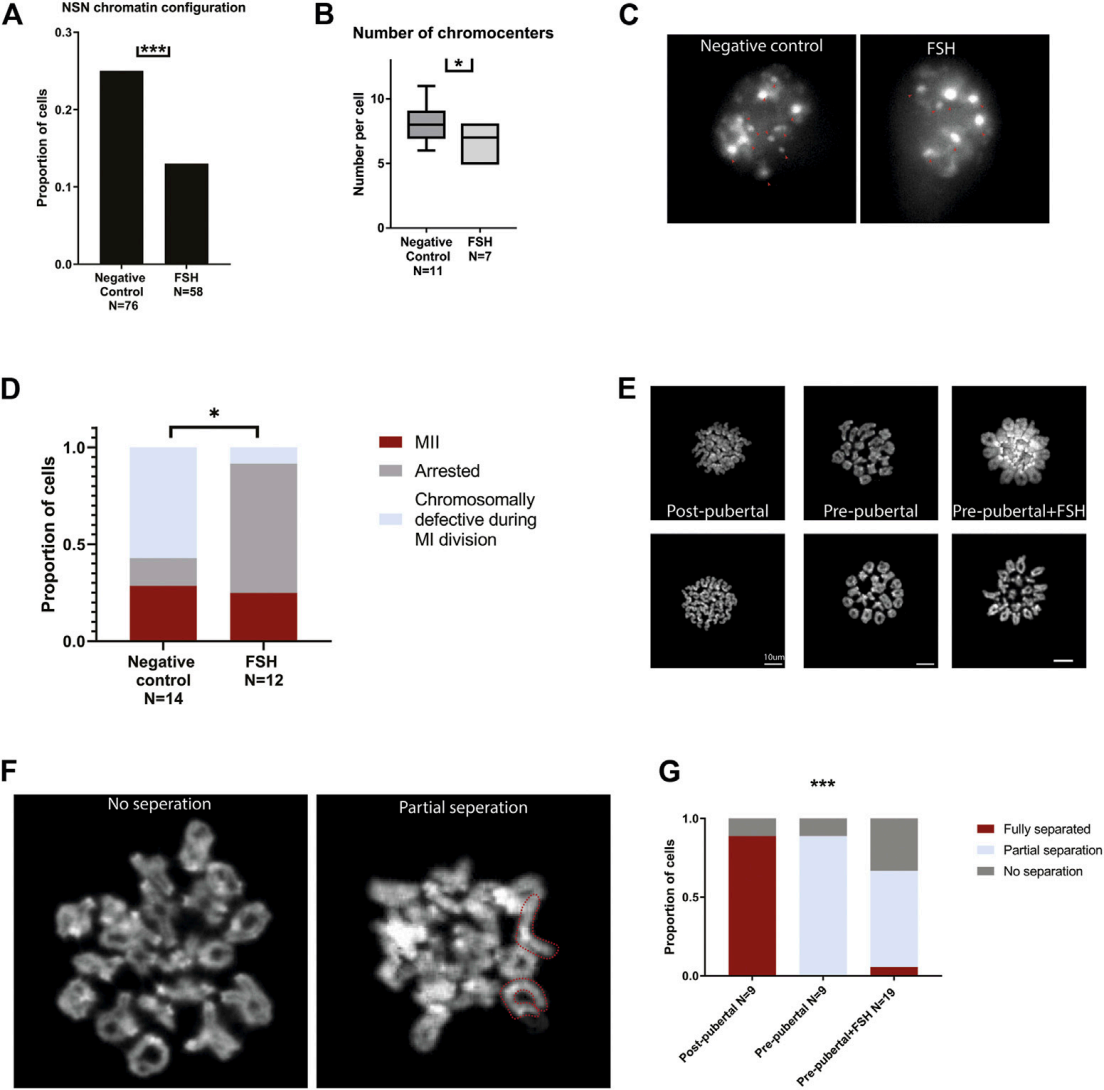
*In-vitro* treatment of pre-pubertal oocytes with FSH initiates chromatin remodeling and modifies meiotic and cellular function: Pre-pubertal oocytes were collected and cultured in FSH supplemented medium, and chromatin organization and *in-vitro* maturation efficiency were assessed. **(A)** GV oocytes chromatin condensation was measured after exposure to FSH. Treated oocytes presented an improvement in chromatin configuration, - NSN configuration was less common in this group (z test *p* = .00019, N_animal_ = 4). **(B–C)** Among NSN-configured oocytes - FSH treated oocytes presented better chromocenter clustering (MW test *p* = .0141, N_animal_ = 4). **(D)** In the treated group, most of the oocytes did not proceed to MII, and most of the MII oocytes presented proper segregation. In the control group, abnormal segregation was more common, and fewer oocytes were arrested in MI after IVM. (Chi-square test *p* = .0106, N_animal_ = 4). MII metaphase chromosome spreads were performed on oocytes from post-pubertal oocytes, pre-pubertal oocytes, and pre-pubertal oocytes after prolonged culture in FSH. While post-pubertal oocyte homologs separation was successfully accomplished, most of the pre-pubertal oocytes presented separation defects, independently of FSH treatment **(E)**. Separation defects were divided into partial separation and no separation phenotypes. Partial-separation was defined with one or more single homologs observed in the spread. Examples of no-separation and partial separation phenotypes are presented with fully separated chromosomes depicted in red **(F)**. The separation phenotype was significantly different between post-pubertal and pre-pubertal oocytes **(G)**, with loss of separation function in pre-pubertal oocytes (Chi-square test *p* = .0007, N_animal_ = 6). FSH treatment mildly improved this phenotype. A slight increase of partial separation appearance was observed in treated pre-pubertal oocytes (Chi-square test *p* = .0003, N_animal_ = 6).

Following our observations of FSH involvement in changing pre-pubertal oocytes histone PTMs, we further studied the effect of FSH treatment on pre-pubertal oocytes chromatin and cellular functions. Pre-pubertal mouse oocytes were collected and exposed in culture for 24 h to FSH-supplemented medium. To examine their meiotic function, cultured oocytes underwent standard IVM culture with FSH followed by oocyte maturation assessment (see Methods). To evaluate the effect of this FSH treatment on oocyte maturation, chromatin organization and cellular functionality, we used the same parameters used above for the comparison of pre-pubertal and post-pubertal oocytes. GV oocytes presented improved chromatin-organization features. Similar to pubertal transition, FSH treatment resulted in an increased conversion to SN chromatin structure (Figure 7A) and a decreased number of chromocenters in NSN oocytes. (Figures 7B, C). We then studied how FSH exposure affects *in-vitro* maturation rates in pre-pubertal oocytes. Interestingly, there were some changes in meiosis progression in treated oocytes after IVM. The rates of proper MI completion were comparable in the control and experimental groups. However, in FSHtreated pre-pubertal oocytes that progressed past MI entry, the ratio of properly matured oocytes was 6-fold higher than in the untreated group (Figure 7D). This gap is explained by the increased incidence of oocytes that remained arrested before entering MI metaphase in FSH treated oocytes. This result might indicate that FSH exposure does not enhance proper MI completion but regulates correct entry into it. MI arrested oocytes were either GV or prophase I arrested, without finding any significant difference in the distribution of this subgroup.

To evaluate the effect of FSH on chromosome structure in pre-pubertal oocytes, we performed Metaphase II chromosome spreads and evaluated the structural state of chromosomes in oocytes at this stage with or without FSH treatment. Normal metaphase II chromosomes are jointed only at the centromeres, which are easily recognized in Hoechst staining by their dense staining signal (Chambon et al., 2013). Post-pubertal oocytes served as a positive control, presenting homologs separation in 89% of the cells. Untreated pre-pubertal oocytes failed to achieve successful arm separation with homologous chromosomes remaining entangled at metaphase II, suggesting a possible defect in crossover resolution (Figure 7 D-G). A small increase in partial separation phenotype was observed in FSH-treated oocytes compared to untreated pre-pubertal controls (Figure 7 D-G). These findings point to a partial improvement in meiosis progression in the presence of FSH. However, other elements and factors may be necessary to establish cellular competence and higher maturation rates.

## Discussion

It is well known that puberty is one of the biggest changes in a gamete’s life cycle (Wasserzug-Pash and Klutstein, 2019). Nevertheless, more information is needed about the effect of puberty on the oocytes and about the biological features that distinguish post-pubertal oocytes from pre-pubertal ones. Here, we characterized an epigenetic aspect of this transformation and revealed a chromatin remodeling event that occurs during puberty. Previous reports (Kusuhara et al., 2021; Karavani et al., 2021) (Karavani et al., 2019) showed an increase in IVM rates and a decrease in meiotic defects of oocytes matured *in-vitro* taken from post-pubertal as compared to pre-pubertal animals. We have confirmed these reports in another mouse strain and showed that chromatin configuration is perturbed in pre-pubertal oocytes, and more likely to remain NSN. NSN oocytes in this age group also presented an undeveloped organization, observed by having more chromocenters. Chromatin remodeling was apparent also when looking at histone modifications. Pre-pubertal oocytes presented an increase in histone acetylation, as well as a decrease in histone methylation compared to the post-pubertal group. Low histone methylation levels appeared to be strictly regulated and not easily modified by chemical intervention in pre-pubertal oocytes. However, exposure to FSH elevated methylation levels and initiated chromatin remodeling in those cells, accompanied by an altered meiotic function. Presumably, this occurred *via* the effect of FSH on granulosa cells surrounding the oocyte (Yu et al., 2022) (Bhartiya and Patel, 2021), known to express receptors for FSH, but this needs to be further demonstrated. Importantly, the effect of FSH on pre-pubertal oocytes is only partial. FSH exposure directs the oocyte toward a post-pubertal-like phenotype but fails to fully accomplish this progression. The partial effect of FSH on pre-pubertal oocytes can be demonstrated in two observations that could be related. First, in contrast to post-pubertal oocytes, which had lower histone acetylation levels than before puberty (Figure 2E), FSH treatment produced an increase in histone acetylation (Figure 6D). The second observation showed that although the pre-pubertal GV oocytes present improved chromatin organization after FSH treatment, once meiosis is resumed it is usually not completed (Figures 7D–G). Previous studies on histone acetylation in oocytes showed that de-acetylation is required for proper meiotic segregation (Ma and Schultz, 2013) (Sui et al., 2020) (Chen et al., 2021). Thus, the acetyl-saturated histone profile of pre-pubertal oocytes treated with FSH pattern might present as an obstacle to their meiotic activity. Future experiments will address this possibility.

FSH exposure alters histone modifications of pre-pubertal oocytes but fails to achieve the final product of meiotically competent mature oocytes. The partial effect of FSH on pre-pubertal oocytes indicates that additional factors are involved in this process. These factors could originate either from the systemic changes that occur in the growing body, or specifically from the hypothalamic–pituitary–gonadal axis (HPG axis) that activates puberty. Knockout of G-protein coupled receptor (GRP54), Kisspeptin receptor, inhibits HPG activation and puberty, and knock-out animals do not go through HPG axis activation and remain pre-pubertal. To have an isolated view on puberty, we investigated previous reports on GPR54 knockout mouse oocytes (Chan et al., 2009) (Seminara et al., 2003). Gonadotropin injection to these animals bypasses the GRP54 pathway, and activates the ovary directly, generating ovarian puberty-like function. When knockout naïve adult animals are primed with gonadotropin treatment, they produce euploid oocytes at similar rates to their WT littermates (Chatzidaki et al., 2021). Interestingly, in advanced age, gonadotropin priming produces less age-related aneuploidy in the GPR54KO animals than in their wild-type age-matched littermates. The ability to accomplish successful meiotic segregation in the vulnerable context of aged oocytes, is suggestive of a critical effect of the first hormonal exposure on oocytes chromatin, that in combination with other pathways may work together to achieve full meiotic competency in oocytes. This avenue should be further explored in future experiments, thus expanding the present study with the inclusion of more HPG players such as LH in the experimental setup.

Clinically, our results are of importance. We showed that exposure of arrested oocytes to FSH has importance to events that occur before meiotic resumption. While it is common practice to use FSH and LH in *ex-vivo* protocols in fertility preservation procedures, treated oocytes are already extracted from the ovarian context and therefore are no longer meiotically arrested. The necessary step of chromatin condensation and organization in the meiotically arrested oocyte, which enables better meiotic function after meiosis resumption, is so far neglected. CAPA-IVM, an IVM protocol that includes pre-IVM culture of small follicles has been in use, and some reports suggest that it could improve IVM efficiency. There is a real possibility that giving oocytes more time before meiotic entry will improve the chromatin status of oocytes, thus accounting for the improvement of CAPA-IVM vs. standard IVM protocols (Sanchez et al., 2019) (Zhao et al., 2020). Following our work, common IVM practices should be re-examined and modified in a way that will enable better reproductive outcomes.

Moreover, our findings may have importance in understanding the processes which occur upon transplantation of ovarian tissue to patients in remission. Some patients that underwent OTC before puberty choose to transplant back the tissue years later, after puberty. The oocytes found within the tissue are all pre-pubertal, but are transplanted back into a body, that has high levels of FSH. It is tempting to hypothesize that chromatin organization and maturation ability of these oocytes may be reassuringly normal after exposure to FSH, but this still needs to be experimentally and clinically examined (Hornshoj Greve et al., 2021) (Shapira et al., 2020).

## Materials and methods

### Animals

RCC-C57BL/6JHsd female mice were used for the experiment. Pre-pubertal animals were used within 2 days of weaning (∼21 days old). For the pot-pubertal group we used 7–13 weeks-old mice. The experiment was approved by the institutional ethics committee, approval number: MD-21-16731-4. All the mice were held at the Hebrew University AAALAC-accredited and NIH-accredited SPF facility. Anesthesia was administered to animals before euthanasia. Animals were anesthetized by injection of Ketamine and xylazine 200 mg/kg BW and 10 mg/kg BW IP. Euthanasia was performed by cervical dislocation.

### Mouse oocyte *in-vitro* maturation

After euthanasia, ovaries were collected and dissected in L-15 medium (011151A) supplemented with 200 µm IBMX (I7018) to prevent meiotic progression. GV oocytes were collected under a binocular using a Stripper (MD-MXL3-STR-CGR). After collection, the oocytes were transferred into α-MEM medium (22561021) supplemented with IBMX covered with Mineral oil (M8410-1l) to prevent evaporation, for a recovery time of 25 min at 37° in a 5% CO_2_ incubator, and then washed in IBMX free α-MEM medium to initiate meiosis. The oocytes were incubated for 24 h [timing was determined according to (Ishikawa et al., 1992)] in α-MEM under oil after which they were stained by Hoechst to examine the entry into the second meiotic division.

### Mouse oocytes collection for *in-situ* immunofluorescence

Oocytes were retrieved with no hormonal or any other priming. After euthanasia, ovaries were collected and dissected in M2 medium (M7167). GV oocytes were collected under a binocular using a Stripper and washed in hyaluronidase (H4272-30MG) to remove granulosa cells and acidic Tyrode’s Solution (T1788) to remove the Zona Pellucida. The oocytes were fixed using PFA 4% (15710) for 20 min, and then quenched in PBS supplemented with 10 mM glycine and 1% BSA.

### Chromosome spreads and immunofluorescence

GV oocytes were collected from ovaries as above and matured for 17.5 h for second meiosis metaphase as described above. Matured oocytes were identified by polar body extrusion. They were washed in M2 medium and then in acidic Tyrode’s Solution to remove the Zona Pellucida. 15 min before the desired time for chromosome spread, oocytes were placed in hypotonic solution, composed of FBS (F7524) diluted in water in 1:1 ratio. Oocytes were then spread in spreading solution (1% PFA buffered to 9.2 pH, supplemented with .15% Triton X-100 and .03% DDT) on Superfrost plus slides (32090003).

### *In situ* immunofluorescence

Permeabilization was performed using .01% Triton X-100. Cells were cultured in PBS containing 5% BSA for blocking, and primary and secondary antibodies were diluted in .1 Tween 20 (P9416)/PBS containing 5% BSA. The immuno-stained cells were mounted in Vectashield (H-1000) mounting medium containing 80 nM of Hoechst (33342), and sealed on a slide using an Imaging spacer (GBL654002).

### Antibodies

For heterochromatin staining we used antibodies for H3K9me2 (ab1220, concentration of 1:400), H3K27me3 (ab205728, 1:50) H4K20me1 (ab9051, 1:500). For euchromatin markers we used antibodies for H3K27ac (8173s, 1:100) and H3K4me3(11960s, 1:50). Active transcription was measured using an antibody for RNA polymerase II CTD repeat YSPTSPS phospho S5 (ab5131, 1:100). To track retroviral activity and transcription regulation loss we used antibodies for L1-ORF1p (ab216324, 1:100), and Dicer (ab167444, 1:200). GFP expression was measured using anti-GFP antibody (Rockland 600-101-215, 1:250).

### Imaging and quantification

Oocytes were imaged using either Ti-Eclipse Nikon system, with an Andor Zyla nsc05537 camera, or Zeiss LSM710. For each experiment, all the cells were imaged under the same settings.

Every cell was imaged at multiple planes. To analyze the images quantitively, a projection of maximum intensity was created. To measure staining intensity, a measurement was taken from the region of interest (ROI). The ROI was defined by the target of the antibody. For nuclear staining (for example, histone modifications) the ROI was defined by positive Hoechst region, and for cytoplasmic staining (retrotransposons, Dicer) by the cell’s borders. For each cell, we also took a background measurement, from a region outside the ROI. For chromatin staining background, we measured inside the oocyte’s cytoplasm, and for cytoplasmic staining-outside the cell. The intensity score was generated by dividing the intensity measurement in the ROI by that of the measurement from the region samples as background.

### qRT-PCR

After euthanasia, ovaries were collected and dissected in M2 medium (M7167) as above. 100 GV oocytes were collected and washed in hyaluronidase (H4272-30MG) to remove granulosa cells, and then washed extensively in M2 until there all the granulosa cells were removed from the oocytes. Oocytes were transferred to TRIzol (15596026) and RNA was purified using Zymo research microprep kit protocol, including DNase treatment (ZR-D7005). Reverse transcription was performed using iScript™ Reverse Transcription Supermix for RT-qPCR (1708891) according to the manufacturer’s recommendations. iTaq™ universal SYBR^®^ Green Supermix (1725124) was used for amplification reactions. Quantitive PCR measurements were taken using a CFX96 C1000 BioRad machine.

### Drug and hormone treatments

Ovaries were collected and dissected in L-15 medium supplemented with 200 µm IBMX to prevent meiotic progression as described above. GV oocytes were collected using a Stripper as described above. After collection, oocytes were transferred to α-MEM medium supplemented with IBMX + the desired drug covered with Mineral oil to prevent evaporation, for 4 h for epigenetic drugs [timing was determined according to (Eppig, 1977)], and 24 h for FSH [timing was determined according to (Eppig, 1977)], at 37°in a 5% CO_2_ incubator. Control group cells underwent the exact same procedure without the drug. After the allotted incubation time oocytes were washed in IBMX-free α-MEM (release medium) to initiate meiosis. The release medium also contained the tested drug in the experiment group. 24 h after oocytes were released from IBMX, oocytes were fixed in PFA, permeabilized, and sealed as described above. Drugs in use were: SRT-1720 (18.7 µm, biovision 2772) and FSH (0.1IU/ul, HOR-249).

### Oocyte Electroporation

Ovaries were collected and dissected, and oocytes were isolated in L-15 medium supplemented with 200 µm IBMX to prevent meiotic progression as described above. GV oocytes were washed with acidic Tyrode’s Solution, and transferred into EC-002 electroporation cuvettes containing 100ul of clean L-15 medium for the control group and 50–200 ng/ul, Lonza pmaxGFP (DMC00054) or CMVp-EZH2 [plasmid was reported by the Sartorelli group (Caretti et al., 2004)] plasmid for the experimental group. Electroporation was done in a Nepagene NEPA21 electroporator. The electroporated oocytes were washed briefly and then cultured in IBMX supplemented α-MEM. After 24 h of culture treated oocytes were fixed and stained with reported antibodies.

### Ovarian tissue cryopreservation procedure

The OTC procedure done in our center includes the removal of the entire (complete oophorectomy) or most of the ovary (partial oophorectomy) in a laparoscopic procedure. The tissue is then placed on ice in Leibovitz L-15 medium (GIBCO-BRL, Paisley, United Kingdom) and transferred immediately to the adjacent IVF laboratory for tissue examination and processing. In some of the cases, mostly in younger patients, insertion of a venous access device (port-a-cath) or bone-marrow aspiration is performed following the oophorectomy. In the case of a normal post-operative course, 24 h discharge is customary. Freezing of ovarian tissue was previously described in the literature (Jeong et al., 2012) and performed similarly in our medical center. Briefly, ovarian cortex is carefully separated from the medulla in a specific media using sterile scissors. The tissue is then cut into pieces of 5 × 10 mm, which are transferred to a pre-cooled freezing medium containing 1.5 M dimethyl sulfoxide (DMSO) and .1 M sucrose. Each fragment is then placed in a 2 ml cryovial containing a cryoprotectant medium and processed using a slow freezing protocol in a programmable freezing machine (Kryo 360, Planer, United Kingdom). The frozen vials, each containing five cortex slices, are then stored in liquid nitrogen.

### Clinical data collection

Data was collected from patients’ electronic files and from the IVF laboratory database. Parameters retrieved included demographic and clinical data, cancer diagnosis, age at diagnosis, treatment with chemotherapy and its timing with regard to OTC, OTC procedure-related information (partial or complete oophorectomy) and IVF laboratory data–including ovarian tissue cryopreservation (number of ovarian tissue ampules cryopreserved), oocytes retrieval and the IVM process and outcomes with emphasis given to the assessment of the number of matured oocytes following IVM, overall and per patient IVM rate according to age groups and percent of patient with at least one matured oocyte following IVM.

### Human oocytes *in situ* immunofluorescence

Following ethical approval (approval no. 0019-19-HMO)- cortex dissection and cryopreservation were performed as described above. An initial scanning of the medium looking for large COCs, which were cryopreserved, was performed in the clinical IVF lab. The remaining medium, containing oocytes, was transferred to our lab after informed consent from the patient, and scanned in the lab for the presence of follicles and oocytes prior to disposal. Previous experience in the lab has shown that due to manual handling of the oocytes during OTC, some of the oocytes initiate cell death. These dying cells heavily bias epigenetic marker staining due to an extremely strong signal after staining. Therefore, oocytes were cultured overnight in α-MEM medium at 37°in a 5% CO_2_ incubator. We then fixed the oocytes and looked for DNA fragmentation (by Hoechst staining) and cellular atresia (by visualization of granular appearance inside the oocyte, visible light). Dying oocytes were removed from the pool of stained oocytes. Remaining oocytes were then fixed in 4% PFA the day after retrieval, for 20 min at room temperature and then quenched in PBS supplemented with 10 mM glycine and 1% BSA. Immunofluorescence was performed as described above.

### Statistical analysis

In order to compare between means, either a parametric or non-parametric test was performed: in the case of N > 30 or if a Shapiro Wilk test returned insignificant value, normality was assumed and the analysis was performed using a parametric test (*t*-test for single comparison and one-way Anova for group comparison). Otherwise, a non-parametric test was used (Mann Whitney (MW) for single comparison and Kruskal Wallis (KW) for group comparison). To compare proportions, Z test for two proportions was used. To assume normality, the role of N > 30 or by N*p > 5 was used for each of the compared groups. To compare distributions of phenotypes, Chi test was performed. Statistical analysis was performed using Excel, R and GraphPad prism.

Significance markings in figures were made by the following rules:

*−*p*≤.05,
**−*p*≤.009,
***−*p*≤.0009,
****−*p*<.0001.

## Data Availability

The original contributions presented in the study are included in the article/Supplementary Material, further inquiries can be directed to the corresponding authors.
