# Supplementary material for: Pre-pubertal oocytes harbor altered histone modifications and chromatin configuration
Source: Front Cell Dev Biol. 2023 Jan 10;10:1060440. doi: 10.3389/fcell.2022.1060440 (PMC9871384; doi:10.3389/fcell.2022.1060440)
Supplement: Supplementary file 1 [file Table1.DOCX]

Supplementary Table 1:

|  | N | Age at OTC* | Chemotherapy before OTC | Number of oocytes* | GV after 24 hour culture* |
| --- | --- | --- | --- | --- | --- |
| Pre-pubertal | 7 | 9.1±2.47 | 57% | 4.57±3.28 | 50.86%±24.5% |
| Mature | 11 | 19±4.2 | 27% | 5.63±2.99 | 32.73%±34.1% |

*Calculated mean and 95% confidence interval.
