# Supplementary figures and images for: Pre-pubertal oocytes harbor altered histone modifications and chromatin configuration
Source: Front Cell Dev Biol. 2023 Jan 10;10:1060440. doi: 10.3389/fcell.2022.1060440 (PMC9871384; doi:10.3389/fcell.2022.1060440)

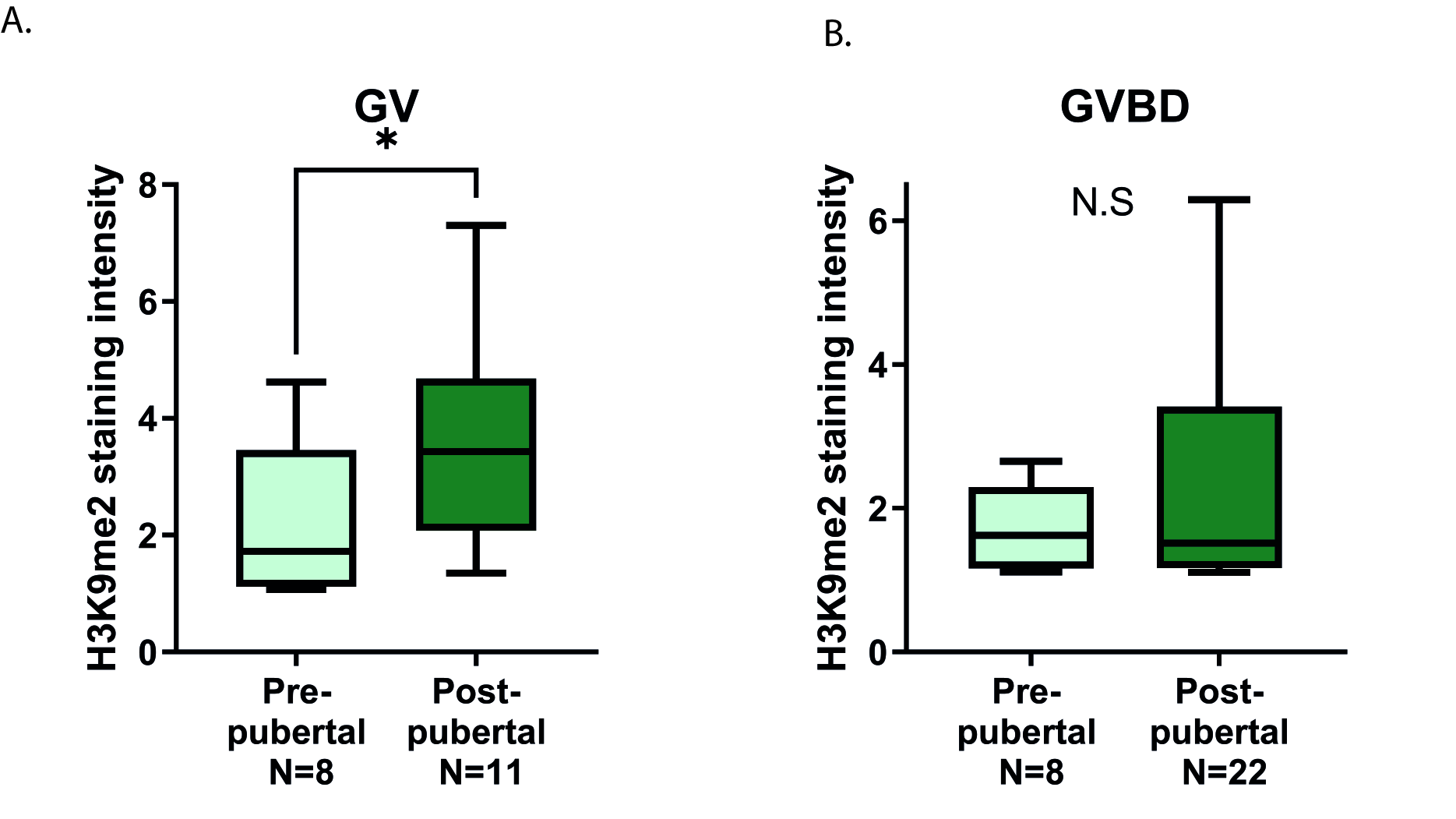

Supplement: Supplementary file 2 [file Image3.TIF]

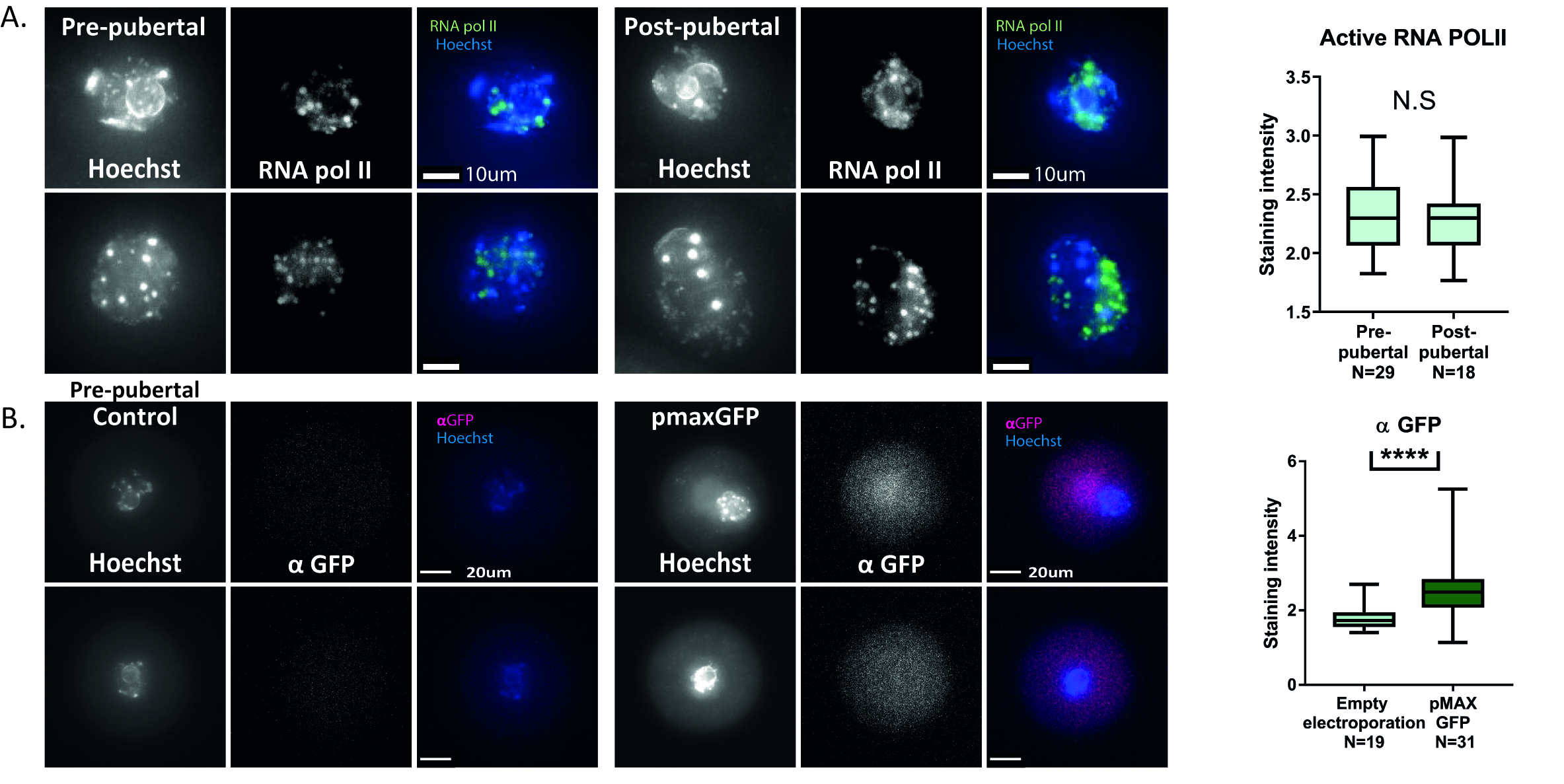

Supplement: Supplementary file 3 [file Image4.TIF]

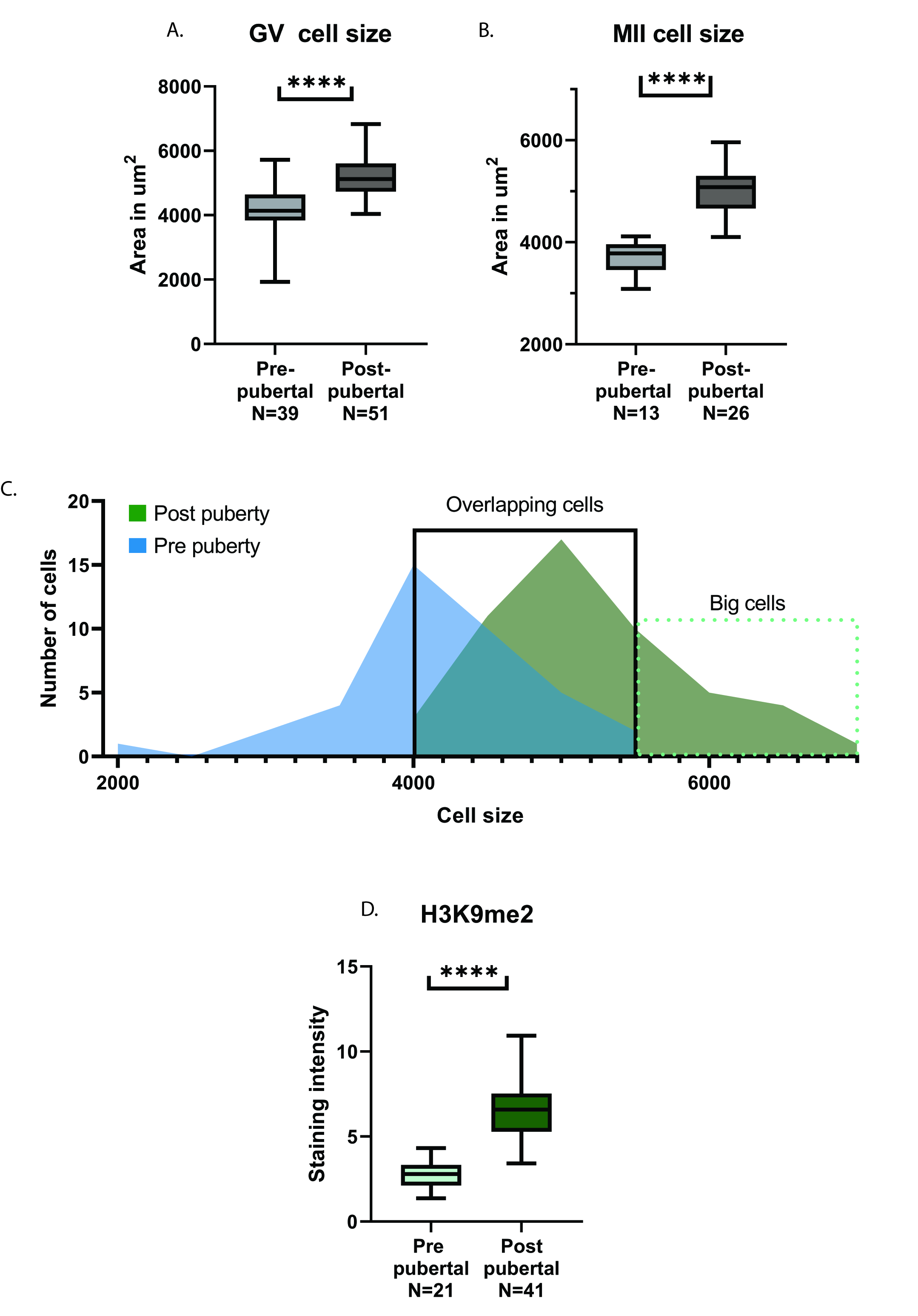

Supplement: Supplementary file 4 [file Image2.TIF]

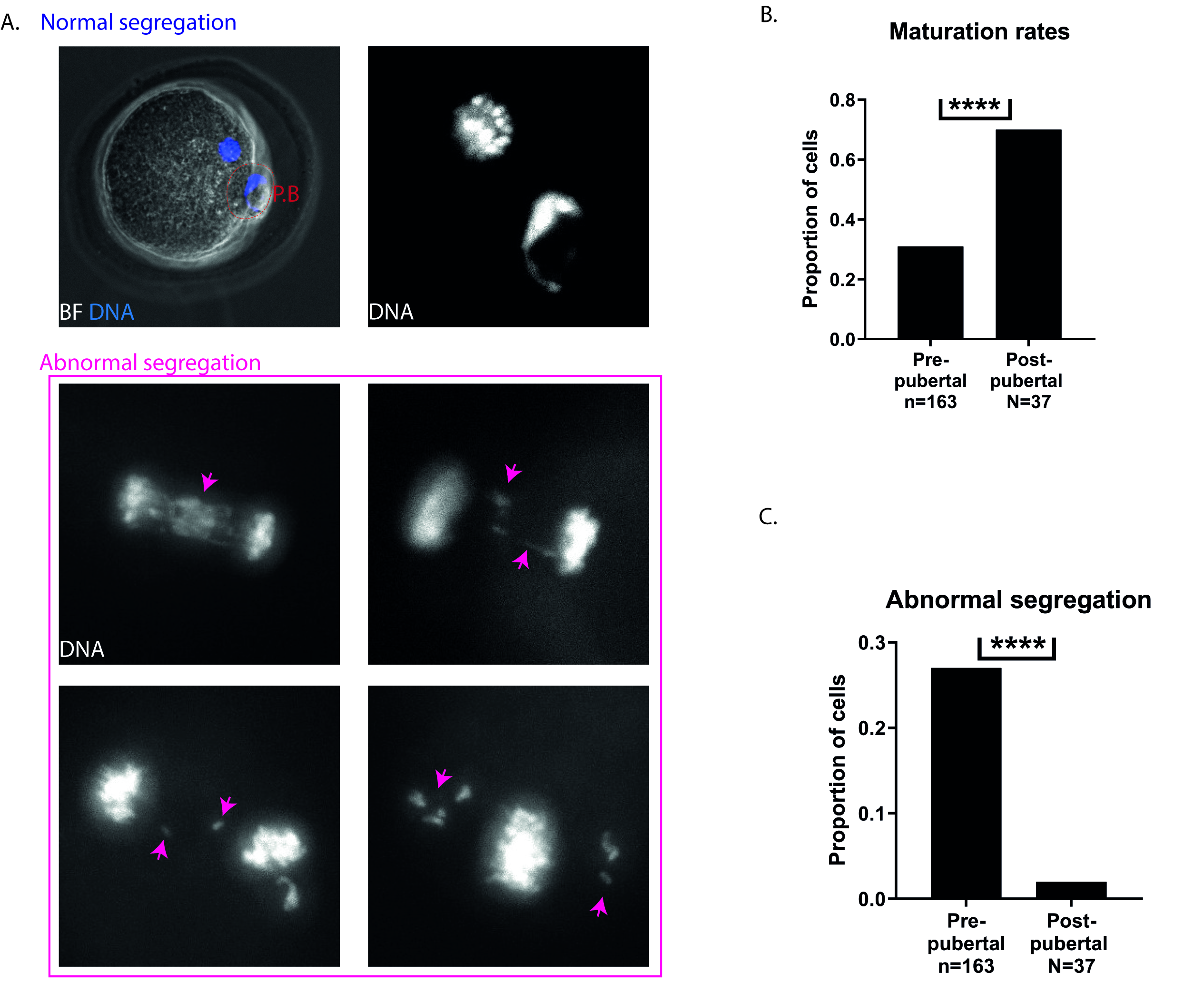

Supplement: Supplementary file 5 [file Image1.TIF]
